# Perceptions and Significance of Long Covid Diagnoses From the Perspectives of Children and Young People With Long Covid, Their Parents and Professionals

**DOI:** 10.1111/hex.70318

**Published:** 2025-06-05

**Authors:** Alice Faux‐Nightingale, Benjamin Saunders, Claire Burton, Carolyn A. Chew‐Graham, Glenys Somayajula, Helen Twohig, Victoria Welsh

**Affiliations:** ^1^ School of Medicine Keele University Keele UK

**Keywords:** adolescent, child, Covid‐19, diagnosis, general practice, Long Covid, qualitative methods

## Abstract

**Introduction:**

Long Covid, the patient‐preferred term, describes symptoms persisting after an acute Covid‐19 infection. Understanding the importance and meaning of a Long Covid diagnosis to children and young people (CYP), their families and professionals associated with their care can give insight into the way that these diagnoses are used across these groups to support care and needs of the patient. This study explores the meaning and importance of a Long Covid diagnosis from the perspectives of CYP with Long Covid, their parents and relevant professionals.

**Methods:**

CYP and their parents or carers were invited to interview following participation in an initial cohort study. Professionals with experience working with CYP with Long Covid were invited to participate in a focus group. Interviews were carried out with four CYP with Long Covid (all female, aged 10–17 years); parents were present at three interviews. Seven professionals with experience in the care of CYP or Long Covid participated in one of two focus groups. Data were analysed thematically using constant comparison techniques.

**Results:**

The three main themes presented are as follows: the importance of receiving a diagnosis, diagnosis facilitates access to support and perspectives of discordance between family and professionals. The diagnosis of Long Covid has different meanings and significance for parents and professionals. Families described the diagnosis as a legitimisation of their experiences and a way to access support, but professionals questioned some of the ways families use the diagnosis, focusing instead on appropriate treatment according to CYP's needs.

**Conclusion:**

For families, Long Covid diagnoses are important for validating and legitimising symptoms, removing uncertainty, and supporting access and participation, particularly in school. While these uses differ from those of professionals, understanding the importance of a Long Covid diagnosis to families may ensure effective communication, negotiation of an acceptable management plan, and ongoing support for this group.

**Patient or Public Contribution:**

Patients and the public contributed throughout this project and had input on the study design, topic guides, and dissemination of findings.

## 1 Introduction

For many children and young people (CYP), severe acute respiratory syndrome coronavirus 2 (SARS‐CoV‐2) infection, the cause of Covid‐19, results in a short‐lived, mild illness [1]. For some CYP, however, symptoms persist; a condition commonly called Long Covid [2]. The estimated prevalence of this varies depending on the definition/s and the population used [3], but has recently been estimated to be 23.36% [4]. Long Covid is a multisystem condition and, in CYP, includes symptoms such as fatigue, breathing difficulties, headaches, myalgia/arthralgia, sleep disturbance, rhinorrhoea, coughing, anosmia/dysgeusia and sensory problems [5, 6, 7]. The combination of symptoms can vary between individuals, and those with Long Covid may experience varying symptoms at different time points. These symptoms can substantially affect CYP, impacting their daily routine and activities, as well as school attendance [8, 9]. Long Covid is a diagnosis of exclusion [7]—there is no single test, and alternative explanations must be ruled out before the diagnosis is applied. The lack of a universally accepted definition at the outset has created challenges in research, in clinical practice and for patients attempting to obtain a diagnosis [10].

Diagnostic labels serve many different functions, and their utility may be different for clinicians and patients. For clinicians, a diagnosis is a way to classify patients according to their presentation and needs, and it supports appropriate clinical decision‐making processes to take place, allowing healthcare professionals (HCPs) to tailor treatment to the patient [11]. The diagnosis acts as a key point of interaction and information exchange between the public and professionals who provide care and support within a patient's healthcare journey, although it is important to note that this is variable between conditions; in some cases, a diagnosis may not be possible or may not substantially contribute towards management. Developing a diagnosis is an iterative process which involves the HCP working with the patient to gather information, make sense of that information and interpret it, leading to a working diagnosis [11], a process which varies in time and complexity according to the patient's circumstances.

For patients and the public, diagnoses can have a different meaning. A diagnosis can acknowledge a person's position as a patient who needs care and allocate responsibility for that care to clinicians [12]. Within their medical journey, diagnoses shape people's experiences of being a patient [13], acting as a point of connection between a patient and their HCPs and the healthcare system as a whole. The diagnosis enables access to specialised services [14] and, outside of the healthcare system, support like welfare benefits [15]. Socially, diagnoses hold further meaning. A diagnosis suggests that there is a scientific explanation for a patient's symptoms and, for some, can offer hope that symptoms can be validated, controlled or treated [14]. It legitimises patients' experiences, allowing access to the role of a sick person [12, 15, 16], and can open up opportunities for people to access a collective identity and associated social networks or integrate their diagnosis into their identity [17, 18] and gather information about their illness [15, 19]. On a wider scale, diagnoses can also affect social narratives of health and contribute towards a societal understanding of illness and its causes [12].

While HCPs and patients may, in some ways and in certain contexts, value diagnoses differently, the diagnosis is a point of discussion [20] and forms part of the interface between these groups as patients move through the healthcare system. If a formal diagnosis is required, patients must seek help from an HCP [20]. However, this consultation can be a site of discordance between HCPs' clinical knowledge about disease processes and patients' lived experience [15, 21, 22, 23], and patients can question or reject a HCP's offered diagnosis because individual health beliefs do not align with the suggested diagnosis [15, 22].

In relation to the diagnosis of Long Covid, a variety of terms and definitions continue to be used worldwide [6, 24]. As the pandemic evolved, there were a number of attempts by a variety of organisations to define the term Long Covid, complicated further by a widening variety of alternative terms for Long Covid such as post‐Covid syndrome, post‐acute sequelae of Covid‐19 (PASC) and Long‐haul Covid, with the ICD (International Classification of Disease) equivalent code ‘Post COVID‐19 Condition’ taking almost 2 years to be created [25]. In the United Kingdom, the National Institute for Health and Care Excellence (NICE) suggested that Long Covid comprised both what it termed ‘ongoing symptomatic COVID‐19’ (symptoms from 4 weeks and up to 12 weeks) and ‘Post COVID‐19 syndrome’ (symptoms persisting 12 weeks or more) [6]. However, without the patient‐preferred term ‘Long Covid’ being used as a disease term in healthcare records, this is likely to cause further confusion for patients and clinicians. Although there is now a World Health Organization (WHO) definition [26], parts of the definition remain subjective, and this has not been universally adopted, with the original NICE definition still being used in the United Kingdom and the Centers for Disease Control and Prevention (CDC) in the United States using a different definition again [27]. This is further complicated by a lack of a separate definition for children until much later in the pandemic, with the WHO definition lagging some 18 months behind that for adults [28]. The large UK CLoCk study looking at Long Covid in children used a research definition created through a modified Delphi process [29], which, although stating it was aligned to the WHO definition, is different from both the WHO child definition and the UK NICE definition of Long Covid [29].

The term Long Covid is widely used by patients and clinicians in the United Kingdom. In this paper, we will use ‘Long Covid’ to refer to physical and psychological symptoms that last more than 4 weeks after an acute episode of Covid‐19. Where participants have used an alternative term, this alternative will be retained in quotations.

Understanding the importance and meaning of a Long Covid diagnosis to CYP, their families and professionals associated with their care can give insight into the way that these diagnoses are used across these groups to support care and needs of the patient. As a relatively new diagnostic label, there are many uncertainties that still remain about Long Covid diagnoses and the way that they are perceived and used. Exploring this further could improve HCP–patient communication about Long Covid and patient experiences. This study explores the meaning and importance of Long Covid diagnoses as perceived by CYP with Long Covid and their parents, as well as professionals who care for CYP with Long Covid. We explore the ways that diagnoses are conceptualised and discussed by both groups, considering differences in perception between these groups.

## 2 Methods

This qualitative study was nested within the SPLaT‐19 project (Symptom Patterns and Life with longer Term Covid‐19 in CYP), which was a UK‐based mixed‐methods study (systematic review, cohort and qualitative studies) aiming to provide a picture of the longer‐term effects of an acute Covid‐19 infection, in the context of an established vaccination programme, in CYP aged 8–17 years, residing in the West Midlands of England [30].

The cohort comprised CYP (8–17 years old) identified from 40 GP practices who were invited to complete online questionnaires which recorded incidences of Covid‐19, symptoms and quality of life‐related outcomes over a 12‐month period.

### 2.1 Public and PPIE Input

PPIE was embedded throughout this study from study development to dissemination of findings. A young person, aged 17, was a co‐investigator and has been involved in the development of the study, topic guides and dissemination activities. The National Institute for Health and Care Research (NIHR) Clinical Research Network (CRN) West Midlands Young Research Champion's Group also contributed to the topic guide for the interviews and focus groups (six CYP, all mid‐late teens) and to the analysis and dissemination (seven CYP, all mid‐late teens).

### 2.2 Ethical Approvals

This study received ethical approvals from the East of England—Cambridge South Research Ethics Committee and the Health Research Authority (IRAS: 310580 22/EE/2206).

### 2.3 Recruitment and Sampling of Interview Participants

CYP in the cohort study, who had consented to further contact and reported at least one case of acute Covid‐19 infection with symptoms persisting for more than 4 weeks within the previous 6 months, were invited to participate. Covid‐19 infection was determined either by a positive test result (lateral flow or polymerase chain reaction—PCR) or as self‐reported by the participant, in recognition of the fact that testing was not available during all stages of the pandemic. Participants were not required to have been formally diagnosed with Long Covid, as the aim of the study was to explore issues around diagnosis, including capturing the experiences of people who had experienced prolonged symptoms but who had not presented to healthcare or had not had a diagnosis confirmed.

Sixty‐seven invitations were sent to eligible CYP (or their parent/guardian) via email, along with information about the linked interview study. The research team intended to sample prospective interview participants; however, low interest in the study meant that all CYP who responded were interviewed. Participants were offered a £20 gift voucher to thank them for their participation. CYP younger than 16 years were required to be accompanied by a parent or carer during their interview; CYP aged 16 and above could choose to attend the interview independently. Parents who attended interviews were encouraged to engage in discussion.

### 2.4 Recruitment and Sampling of Focus Group Participants

Professional networks were used to recruit professionals, including GPs, physiotherapists, mental health nurses, consultant paediatricians and teachers who work with CYP or who worked with CYP or families within Long Covid services. Prospective participants were sent a study invitation and information sheet by email. Some focus group participants were known to the research team as colleagues before participation.

### 2.5 Data Collection

Semi‐structured interviews with CYP explored participants' views of having Long Covid and their experiences, if any, of seeking help for their symptoms. Focus groups with professionals involved in the care of CYP with Long Covid explored the professionals' experiences of working with CYP and families with Long Covid and any CYP‐specific training they had received.

Topic guides were developed with the study PPIE group and used flexibly in the interviews and focus groups to respond to participants' words. These topic guides were modified iteratively as data generation and analysis progressed to respond to topics of interest raised in conversations. Topic guides are included in Supporting Information 1.

Interviews took place between December 2022 and April 2023. In England, Long Covid clinics for CYP referred from primary care were funded, commissioned and then established in some localities in 2021 [31]. While these clinics were available during the time of data collection for this study, in 2024, the national Long Covid programme was delegated to Integrated Care Boards (ICBs) [32] and since then, many Long Covid services have been de‐commissioned or merged into existing services.

Interviews occurred on university premises (three) or using Microsoft Teams (one), according to participants' preferences. The interview length was guided by the participant, and rest breaks were incorporated as necessary. All interviews were conducted by A.F.N., a female qualitative researcher.

Focus groups took place virtually using Microsoft Teams. These sessions were facilitated by A.F.N. and B.S. (PhD) (female and male qualitative researchers) and supported by G.S. (female academic clinician with experience of working with CYP) to document observations.

Interviews and focus groups were recorded, and audio/video recordings were professionally transcribed.

### 2.6 Data Analysis

Data collection and analysis were carried out concurrently, which contributed to the development of themes and later the collection of data. Data were analysed thematically using the constant comparison method [33]; each transcript was analysed separately, and then the analyses were mapped onto other transcripts to compare views across the participants.

Transcripts were inductively coded manually by A.F.N., using NVivo 14 software. Themes were developed through a process which involved coding individual transcripts, mapping codes across interview and focus group data, which were then used to develop preliminary themes, and then further comparison across the whole dataset to refine and develop the preliminary themes. B.S. examined the codes and emerging themes and checked them.

Two authors, A.F.N. and B.S., developed preliminary themes together. Themes were considered in relation to existing theory and clinical practice with a multidisciplinary team (all authors) and were refined and finalised by the group.

The interviews and analysis of this study primarily explored the lived experiences and care needs of CYP with Long Covid. The significance and meaning of a Long Covid diagnosis were not the main focus of the study. Still, they were identified as a significant theme during analysis and were highlighted as an area for further, more detailed analysis. Findings pertaining to the experiences and care needs of CYP with Long Covid were published separately [8].

A.F.N. and B.S. are qualitative health researchers; C.B., C.A.C.G., H.T., G.S. and V.W. are academic general practitioners (GPs) who have experience working with families and CYP with Long Covid. A reflexive approach was taken where all researchers considered and acknowledged the influence of their own experiences and backgrounds in their interpretation of the data.

## 3 Results

**Figure 1:**
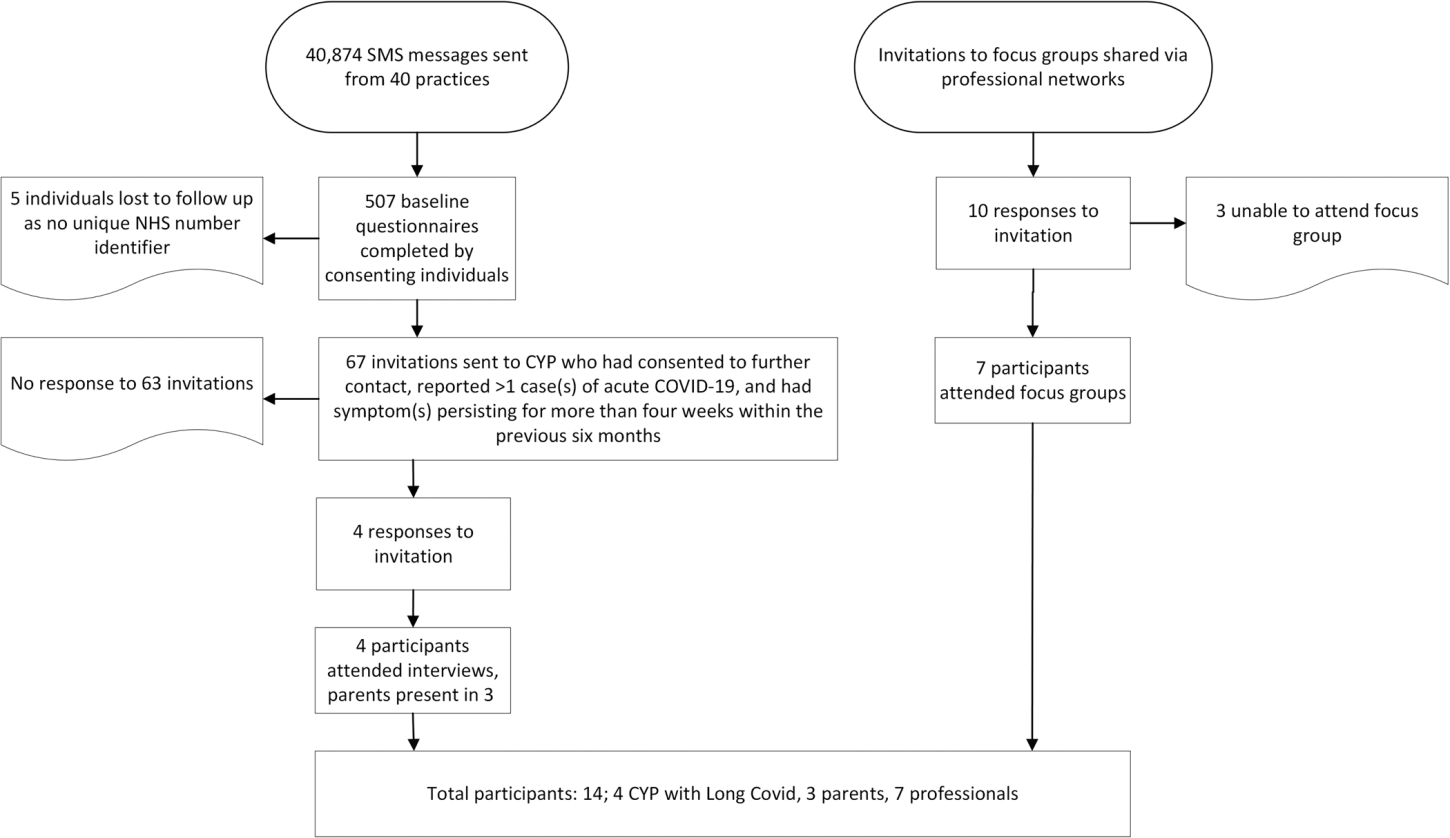
Recruitment flow chart.

Four CYP expressed interest in the study (4 females, aged 10–17) and all participated in an interview ranging from 43 to 75 min, see Figure 1 for a flowchart of the recruitment process. Participant characteristics are presented in Table 1. Three interviews were dyadic and included a parent (all female). Interviews took place between December 2022 and April 2023; at the point of interview, three CYP were still experiencing persistent symptoms, while one had fully recovered. CYP described a range of symptoms: all experienced fatigue and headaches, while some mentioned anxiety, breathing difficulties or dysfunctional breathing, and loss or altered smell or taste. Three CYP experienced symptoms that they reported had affected their ability to attend school full time [9]. Although all participants described experiencing prolonged symptoms following acute Covid‐19, only two participants had received a Long Covid diagnosis at the time of interview; both of these participants had accessed private healthcare. A third participant had been offered an appointment at a Long Covid clinic but had been unable to attend due to travel difficulties.

**Table 1:** Interview participant characteristics.

Participant identifier	Age at interview (years)	Sex	Ethnic group	Accompanied by a parent	Sought treatment for Long Covid	Received Long Covid diagnosis at the point of interview
01	14	Female	Mixed: White and Black Caribbean	Yes	Yes	Yes
02	12	Female	White: English, Welsh, Scottish, Northern Irish, British	Yes	No	No
03	10	Female	White: English, Welsh, Scottish, Northern Irish, British	Yes	Yes	No
04	17	Female	White: English, Welsh, Scottish, Northern Irish, British	No	Yes	Yes

Ten professionals expressed interest in the focus groups and seven (3 M, 4 F) attended one of two groups (one group had five participants and the other, two). Focus group participants worked in a range of settings, varied across the groups and were geographically spread across the United Kingdom. The first focus group was comprised of two paediatric consultants (both with clinical experience with CYP with Long Covid), one nurse consultant (Child and Adolescent Mental Health Services), one physiotherapist (adult pain and Long Covid services) and a primary school teacher. The second focus group was made up of a physiotherapist (Children's Long Covid hub^1^) and a GP.

Although CYP participants were not required to have been given a clinical diagnosis of Long Covid to be included in the study, families commonly raised Long Covid diagnoses in the context of clinical care and social and educational settings. Within interviews, CYP and parents were encouraged to express their views openly and individually, although joint constructions of their experiences also took place. Parents generally talked in greater detail about their experiences of pursuing health care, treatment, the role of a diagnosis in their child's life and the impact of symptoms on family life, while children generally discussed their lived experiences of Long Covid and the impact of symptoms on them as an individual, their education and hobbies. The importance of a diagnosis was not commonly described by the CYP interviewed, in contrast to parental contributions and consequently most of the included statements are from a parental perspective.

Across the dataset, CYP, parents and professionals described perceptions of Long Covid diagnoses, ascribing different levels of meaning and importance to the diagnosis and the way that it was used to facilitate care and support for CYP. Three key themes were developed from the data: The importance of receiving a diagnosis, diagnosis facilitates access to support and perspectives of discordance between family and professionals.

### 3.1 The Importance of Receiving a Diagnosis

For families, finding information about Long Covid and identifying with the symptoms, or self‐diagnosing, provided relief and legitimisation of their experiences. One participant described the sense of legitimisation that she felt on seeing a feature on Long Covid on the news:

‘They [were] talking about Long Covid in children on BBC news […] That was like a huge thing seeing it on telly[…]t was nice because like at the beginning in the main bad points of it I'd no idea, so then when it was coming, not only the thing that it was “Oh, that is what I have,” but also people actually like considering it as a thing it was great.’ [sic]

04, female, aged 17

For families, seeking medical advice and receiving a diagnosis facilitated access to information about Long Covid and understanding of CYP's symptoms. Not receiving a diagnosis was associated with confusion about Long Covid and what was likely to happen in the future, with an associated emotional impact on CYP and their parents:

‘Everyone was just guessing. […] It was horrible, I was like “I don't even know how long this is going to be.” And obviously, no one was talking about it so, I was like, am I literally the only person who is like a massive confusion?’

04, female, aged 17

The three CYP who had sought medical advice and treatment for their symptoms described experiencing difficulty receiving a Long Covid diagnosis or information about Long Covid, which they attributed to a lack of clinical knowledge about Long Covid in CYP amongst HCPs:

‘The GP has mentioned [Long Covid] and she said it is probably, but there's no research on it. We can't really say one way or the other.’

Parent of 03

This lack of explanation, or understanding of Long Covid, by HCPs was associated with not being believed:

‘Sometimes it would be nice—it would have been nice for somebody to go, “okay yeah we do believe you.” So that would be good because sometimes you just think that people think, “nah.”’

Parent of 03

In contrast to the families, health professionals described how they thought it was important to consider alternative possibilities to explain the symptoms presented by CYP, particularly considering that many symptoms could be due to an alternative condition, for example, chronic fatigue syndrome or juvenile idiopathic arthritis.

‘I view my role in seeing [children with symptoms] as trying to make a diagnosis of what's going on and ruling out anything significant […] So trying to rule out if they've got a joint pain, have they got JIA [juvenile idiopathic arthritis]? If they've got new onset wheezing that's worse when they run, is it asthma, or is it vocal cord dysfunction, or is it simply deconditioning?’

Consultant paediatrician A

They described being cautious about diagnosing CYP with Long Covid to ensure that other diseases and illnesses are not inappropriately attributed to Long Covid and, thus, treatment for an alternative condition withheld.

‘We have to be just really careful about what we say COVID has caused’

Consultant paediatrician B

Professionals considered a Long Covid diagnosis a burden for individual CYP and their families, and they wished to minimise this:

‘And we do try to normalise things and not create a great medical burden because then you've got to unpick it all and de‐medicalise it and that's really difficult.’

Consultant paediatrician B

Furthermore, HCPs explored complexities associated with the pressures of the pandemic, which were contributing to patients being referred to Long Covid clinics with problems that may not have been a consequence of a Covid‐19 infection.

‘We always see in the fatigue clinic a lot of neuro‐diverse young people who present as being fatigued. Because being neuro‐diverse or being anxious or being depressed are all very exhausting things […] they're now turning up in the post COVID clinics.’

Consultant paediatrician B

### 3.2 Diagnosis Facilitates Access to Support

Parents described how a diagnosis had been a requirement for their child to access necessary services or support at school. In two cases, families described seeking private healthcare to receive a diagnosis to overcome this barrier:

‘They used to basically nag her to go in more until we had to get a private diagnosis […] we've got school saying, [that] I hadn't had a diagnosis so we don't know what's wrong with her, she could be fine. […] So we had to go private, get this diagnosis but it wasn't particularly for any treatment, it was just for the diagnosis just to show school.’

Parent of 01

Conversely, professionals expressed concerns about some parents who they perceived to be overly focused on obtaining a Long Covid diagnosis rather than supporting their child's symptoms. They discussed cases, not specific to but including Long Covid, where they perceived parents had used a diagnosis as a ‘label’ to excuse symptoms or behaviours that may have their origins in more complex home or societal circumstances, for example, fatigue related to deconditioning associated with lockdown measures implemented within the Covid‐19 pandemic, which could be addressed without medical intervention.

‘…people would rather take [medication] and make that be the diagnosis. So yeah, but is it new? Yeah, we've always seen some of that.’

Consultant paediatrician A

‘We've found it's more parents are very keen to label something. So, if a child is struggling with something, rather than facing the kind of reality of it and working their way through it, it is very much a case of it's easier to get a label then it's almost excused.’

Teacher

Despite hesitancy amongst clinicians to provide a diagnosis, parents and professionals did report cases where NHS HCPs and schools were able, or tried, to support the CYP without a formal diagnosis of Long Covid:

‘She'd had a letter from the doctors saying she should be on a reduced timetable.’

Parent of 01

‘School have been fab[ulous]. [03]'s teacher is new to the school, he only joined in September so we had a meeting a couple of days before term. […] We had a meeting before and he wrote down all the things, we put an action plan in place and a care plan. So, they've been absolutely fab.’

Parent of 03

While a Long Covid diagnosis was not explicitly mentioned by most young people, the impact of receiving support, particularly at school, through having a diagnosis was apparent in their narratives. For example, the following participant talked about the positive impact of having taken time off from school and a reduced timetable, adjustments made while she recovered, which were only possible for her through a Long Covid diagnosis:

‘And now that I'm like this, the past couple of days haven't been in school, it's way better than before. If I went in school for 2 days before, I would probably be asleep by now, I just—I feel kind of like refreshed in a way, I feel like it's just comparing from then, I just feel like it's a lot of weight off my shoulders in a way.’

01, female, aged 14

A diagnosis of Long Covid also offered some support to parents who, through the diagnosis, were able to enter and engage with networks of families in similar situations, for example, Long Covid Kids, a charity for CYP with Long Covid and people who care for them^2^. These connections provided information and social support for parents, particularly during difficult periods:

‘We do a Sunday night [session for] parents, just to have a natter with us parents of how it's been. You know, sometimes you have like a really difficult week and sometimes it's nice just to have a chat with other parents what they're in the same situation.’

Parent of 01

Most clinical professionals appeared to be comfortable with uncertainty around the exact aetiology of symptoms and were primarily focused on treating symptoms; however, this was not universal, with one clinician describing using a Long Covid diagnosis ‘label’ strategically. They described how the prevalence of Covid‐19 and a broad definition of Long Covid allowed them to allocate CYP to services with shorter waiting lists, as funding for Long Covid services at the time meant that waiting lists were shorter and care was more easily accessible than through some non‐Long Covid services.

‘In terms of labelling, they have had Covid, I can label them as post Covid, fine, that means they can fit in my service and I will help them. […] we have been hammered and hammered and told the funding's for post Covid, it's not for chronic fatigue so, I am going to label everything as post Covid because it means they can have treatment and I am really sorry, as a clinician, I am going to do anything I can to get my kids what they need.’

Paediatric physiotherapist B, Long Covid clinic

### 3.3 Perspectives of Discordance Between Family and Professionals

There were examples of discordance between parental opinions and the diagnosis received from HCPs. In these cases, instead of legitimisation of experiences, parents described feeling that HCPs' suggestions did not completely represent their child's symptoms and tried to justify why they thought that Long Covid was more appropriate for their child:

‘But some doctors try to put it down to just anxiety, they try to say “oh yeah, it's probably anxiety due to lockdown [Covid restrictions imposed in the UK]” […] She was doing things after lockdown, she was absolutely fine but she didn't have any anxiety in the early stages when she went back to doing things. It was only after she was unable to do things physically that she got the anxiety. One doctor tried to put her on antidepressants, tried to say it was all depression and there was nothing physically wrong with her, which just isn't true.’

Parent of 01

In these cases, parents generally attributed those suggestions to either a lack of knowledge about Long Covid or the lack of time that HCPs had to talk to their children.

‘Because it is true what they say, doctors get a five‐minute snippet and they have to base their diagnosis on that.’

Parent of 03

One parent tried to resolve this discordance by seeking a second opinion from a private paediatrician.

‘We heard about this private paediatrician who specialises in ME and chronic fatigue syndrome, who actually advocates for families who are in similar situations, so I contacted him and explained the situation.’

Parent of 01

However, professionals were sceptical of patients seeking diagnoses through private healthcare:

‘I'm not big on private medicine for children and young people […] certainly it's very difficult to know how good the provision is from private medicine […] I have seen quite a number of young people whose families have sought private therapy or private this therapy or that therapy or another therapy, I'm not sure whether it's doing any good or not.’

Consultant paediatrician B, Long Covid clinic

## 4 Discussion

As increasing research emerges about Long Covid and the way that it can affect individuals and families, it is important to consider how Long Covid diagnoses are perceived and used by key stakeholders.

For parents and CYP, a diagnosis of Long Covid was associated with validation of their experiences, a way to understand the symptoms the CYP had been experiencing and to receive support in clinical, educational and social areas. Existing literature from other conditions suggests that for patients, a diagnosis suggests that there is a scientific explanation for their symptoms and offers hope that symptoms can be controlled or treated [15]. As seen in this study, the diagnosis legitimises patients' experiences, facilitates pathways to treatment and support [12] and opens an opportunity for people to gather more information about their illness [12, 15, 16]. Not having a diagnosis, reinforced by HCPs who reportedly acknowledged knowing little about Long Covid, was associated with uncertainty about what was causing CYP's symptoms and how that would impact them in the future. Similar findings have been found in other Long Covid studies, receiving a Long Covid diagnosis can be a long process, leaving patients in a liminal position regarding legitimisation of their experiences [34], although research into CYP accessing Long Covid services suggests that having access to those services can also contribute to feelings of validation [35], rather than just the diagnosis alone. In contrast to the parents' views, HCPs considered that a Long Covid diagnosis could be a burden for parents and described working to de‐medicalise treatment for patients and their families to reduce this burden. Long Covid diagnoses were not specifically seen as necessary to give the CYP the treatment and support they needed, which was instead provided based on individual symptoms and problems, not on diagnosis. Previous research with parents of CYP with undiagnosed conditions has highlighted the impact on parents of their child not having a diagnosis, contributing to emotional stress and mental health decline. The emotional toll of the uncertainty while caring for a child with Long Covid has been observed in parents and should be considered when supporting this patient group [9]. This study highlighted the positive impact that having a diagnosis can have on CYP and parents [36], despite the different opinions of the professionals in the focus group who were cautious about parents who sought a Long Covid diagnosis and who had seen private clinicians to receive a diagnosis. While the diagnosis facilitated access to specialised services and treatment [14, 20], interviews with families also highlighted the role of the Long Covid diagnoses beyond the healthcare setting, and how it supported CYP accessing support in school, though a formal diagnosis was not required by all participants to receive support, and parents accessing support from social networks. We know that outside of the healthcare system, diagnoses can also support individuals looking for support in areas like schooling, education [37] and welfare benefits [15], and this was echoed by participants in this study who described seeking a diagnosis because they had been unable to access support at school without a clinical diagnosis. Other studies about CYP with Long Covid in the United Kingdom have also described difficulties for CYP accessing formal support at school [38] with variation in the level of responsivity and whether a diagnosis was required to receive support, and there is evidence of cases where a lack of formal diagnosis acted as a barrier to receiving support from school [39], as observed in our findings.

Recent research suggests that HCPs can facilitate CYP with Long Covid accessing education by assisting the school to support CYP [35]. Although professionals in this study highlighted that some Long Covid services do liaise with schools to ensure appropriate support for CYP, this was not the case for the interviewed CYP and no participants described receiving support in this way, with similar variations in support from schools and support via liaison between schools and HCPs have been seen in other studies across the United Kingdom [39]. While a diagnosis fulfils a clinical role for HCPs and patients, it is important to consider that it may hold significance in other sectors and that the absence of a diagnosis may act as a barrier for some patients trying to access necessary support.

Both families and professionals described cases of contested diagnoses. The negotiation of a diagnosis may be contested by both parties [21, 22, 23]. Indeed, this was seen here by some parents who reported that they had challenged HCPs' suggestions, favouring Long Covid as a more appropriate diagnosis, while HCPs described difficulties with parents who seek medical labels for CYP with symptoms that could be treated non‐medically. Discordance here seemed to occur where families felt that HCPs did not have enough time to listen to them or did not know enough about Long Covid, and in some cases, this caused families to seek private healthcare, a narrative familiar to the HCPs in the focus groups but not perceived positively. However, the families' need for the diagnosis was, in some cases, driven by an external need for the diagnosis to facilitate help from other groups, but may also be affected by other external factors like stigma, reported to be experienced by people with Long Covid [40, 41, 42]. We know that, clinically, a diagnosis enables access to specialised services [14]. In this study, while a diagnosis of Long Covid supported access to Children's Long Covid services, it was also used by clinicians to allocate CYP to services with shorter waiting lists and promote access to necessary services. With the more recent closure of specific Long Covid services funded by the national programme, Long Covid diagnoses may be used in different strategic ways to prioritise patients and families offered support and care. Our findings suggest that clinicians, CYP and their parents use and perceive Long Covid diagnoses differently. The diagnosis has different levels of meaning and significance for the different groups, and these understandings contrast. It is important that clinicians are aware of the significance of a diagnosis beyond the clinical encounter and consider the wider importance of the label to the CYP and their family. By understanding factors behind the perceptions of each group, it is hoped that communication between these parties can be facilitated during patient care. It should be noted that these experiences, particularly comments about access to healthcare and support, are specific to the areas in which participants were located. Provision of healthcare varies across the United Kingdom, and CYP in other regions across the United Kingdom with different access to support may have different views of the need for diagnosis or the way it is used.

## 5 Limitations

Although this study included every participant who responded to their invitation to interview, and nearly all focus group participants who expressed interest, the low response rate led to a small sample size with limited diversity of participants, both in terms of demographics, including only interviewing participants who speak English, and geographical location. This will have limited the experiences captured within the study, and these findings may not represent the experiences of CYP of different demographics or those in different regions with access to different services. CYP recruitment may have been limited by participants' awareness of Long Covid, or difficulties associated with the physical demands of participation, and the study may miss the experiences of those with mild Long Covid who have not identified their persistent symptoms as Long Covid, or the experiences of those with severe Long Covid who may have been unable to manage the demands of the interview. Compound pressures across the NHS and similar pressures in the UK education sector during the time of recruitment may also have affected professionals' willingness to participate. These circumstances may also have contributed towards increased discussion of funding and strained services. Some of the professional participants were known to the research team before participating in the study due to the recruitment method used. We acknowledge that this may have affected group discussions as these participants may have felt more comfortable speaking in the session and felt more willing to discuss certain topics in more detail, potentially prioritising or favouring particular areas. Alternatively, HCPs familiar with members of the research team may have felt inhibited in disclosing their opinions, although we note that the views shared and the degree of disclosure did not seem to vary greatly between the participants in the focus groups. The topic of diagnosis was not the primary focus of the study and was only identified as an important theme for closer analysis during the analytical phases of the study and as such, questions in the topic guide explored families' experiences of healthcare and followed up on participants' comments about diagnoses but did not explicitly ask about diagnoses. However, this is also a strength of this study, which showed flexibility and responsiveness to the data; rather than focusing on pre‐determined topics, the insights from these findings are led by and grounded in the data. Within the interviews, parents more commonly discussed the healthcare processes than CYP, who focused more on discussing symptoms and the impact of Long Covid on their daily life, presented in a separate paper [8]. As a result, most of the comments about diagnosis came from parents and, as such, the perceptions of CYP about Long Covid diagnoses are absent in these data. Future research would benefit from investigating the impact of Long Covid diagnoses on CYP, looking at CYP's interpretations of a Long Covid diagnosis and any effects that it may have on them.

## 6 Conclusions

CYP and families, as well as professionals who care for CYP with Long Covid, discussed their experiences of Long Covid in CYP and their perceptions surrounding Long Covid diagnoses. Our findings suggest that these groups attribute very different, and often contrasting, meanings and levels of importance to Long Covid diagnoses, but these perceptions come from different experiences, needs and understandings of what a diagnosis is used for. By observing these differences and considering the needs and experiences of both groups, it is possible to use this understanding to support communication between patients, families and HCPs at this clinical interface, which may ultimately support both groups in caring for CYP with Long Covid.

## Author Contributions

**Alice Faux‐Nightingale:** writing – original draft, formal analysis, data curation, writing – review and editing, methodology. **Benjamin Saunders:** writing – original draft, writing – review and editing, formal analysis, data curation, methodology, funding acquisition. **Claire Burton:** conceptualisation, methodology, funding acquisition, writing – original draft, writing – review and editing, formal analysis. **Carolyn A. Chew‐Graham:** conceptualisation, writing – original draft, funding acquisition, writing – review and editing, formal analysis, methodology. **Glenys Somayajula:** writing – original draft, writing – review and editing, data curation, formal analysis. **Helen Twohig:** conceptualisation, writing – original draft, funding acquisition, writing – review and editing, formal analysis, methodology. **Victoria Welsh:** conceptualisation, funding acquisition, writing – original draft, methodology, writing – review and editing, formal analysis.

## Disclosure

The views expressed are those of the author(s) and not necessarily those of the NIHR or the Department of Health and Social Care.

## Consent

All participants gave informed consent before participating in the study. Those aged 16 and over (one CYP and professionals) completed consent forms, and participants under 16 read and signed an assent/consent form with their parents.

## Conflicts of Interest

C.C.G. is the editor‐in‐chief of Health Expectations, and V.W. is a member of the National Institute for Health and Care Excellence Indicator Advisory Committee. The other authors declare that they have no known conflicts of interest.

## Supporting information

Supporting material ‐ Topic guides.

## Data Availability

The data that support the findings of this study are available from the corresponding author upon reasonable request.

## Endnotes

1 Paediatric Long Covid hubs were made up of several health professionals who were able to directly treat CYP with Long Covid or refer them to other specialist services and clinics.

2
https://www.longcovidkids.org/.
