# Supplementary material for: Perceptions and Significance of Long Covid Diagnoses From the Perspectives of Children and Young People With Long Covid, Their Parents and Professionals
Source: Health Expect. 2025 Jun 5;28(3):e70318. doi: 10.1111/hex.70318 (PMC12138285; doi:10.1111/hex.70318)
Supplement: Supplementary file 1 — Supporting material ‐ Topic guides. [file HEX-28-e70318-s001.docx]

**Supplementary material: Topic guides**

**INTERVIEW TOPIC GUIDE (updated following previous interviews/focus group)**

YOUNG PERSON

- Explore participant’s experience of Long COVID
  - What has Long Covid been like for you? How long have you been experiencing symptoms? What symptoms do you have?
  - How has Long Covid affected your daily life?
  - Has having Long Covid affected your mental health?
  - How has having Long Covid affected your school life? - Attendance; progress; navigating physical space; accessing school/learning
  - Has your family life changed since you had Long Covid? - care needs; impact on siblings
  - Do you have any hobbies and interests? Has having Long Covid affected how you can engage with them?
  - Has Long Covid affected your friends and relationships?
- Have you sought healthcare or support for your Long Covid? - Which professionals were involved in giving support; In healthcare, was this NHS/Private?; Did they receive support at school?
- What has helped you with your Long Covid? What else might help?
- Have you been able to access information about Long COVID in your age group?
- What information have you/would you find helpful?
- Have you received a Long Covid diagnosis? (How/when did they get the diagnosis?)

PARENT / GUARDIAN

- What is your experience of being a parent/carer of CYP with Long COVID?
- What impact has Long Covid had on the wider family? - Impact on relationships; Impact on working life
- Do you have any concerns for the future?
- Have you sought or received any support to help with your child’s Long Covid? - Thoughts on how support can be improved

**FOCUS GROUP TOPIC GUIDE (updated following previous interviews/focus group)**

Explore participants’ experiences of supporting children and young people (CYP) who have been affected by Long COVID, specifically:

- What experiences have participants had of working with CYP with Long-COVID? (Context: What age groups do participants primarily work with?)
  - E.g. How common are LC presentations in primary care among CYP and adults?
  - GP -How has LC been managed in your practice?
  - Physio - Network of therapists working with CYP post-covid: Are you able to elaborate a little about how/why the network came about, and the support you offer each other?
- Explore experience of common presentation of Long COVID in healthcare settings: symptoms, duration etc.
- Perceived impact of Long COVID on CYP’s daily lives/ activities/ school etc.
- Conversations with CYP and parents about management of symptoms; time off from school etc.
  - E.g. Advice re medications/ pain relief, physical activity, sleep etc.
  - Validating symptoms? Providing reassurance?
  - Have CYP/parents talked about any other services they have accessed for support with their long COVID? E.g. private healthcare/other long-COVID groups?
- Explore similarities and differences with management of other conditions with similar symptoms e.g. chronic fatigue, respiratory conditions etc. (in both healthcare and education settings)
- Explore views on the importance or otherwise of diagnosing LC:
  - What is the significance of diagnosis of LC for referral or management?
  - Explore views on importance of diagnosis from patient perspective
  - GP - What role does a GP play in the diagnosis of LC?
  - Non-doctors - As a therapist do you diagnose LC at all? Have you worked with any children who have similar symptoms to LC but who may not have a diagnosis?
  - How confident do you feel in diagnosing LC?
  - Explore difficulties in disentangling LC symptoms from overall impact of pandemic/ lockdowns on CYP.
  - What are your thoughts about the term long-Covid? Seen some use of post-Covid, wondered if you have a preference and why?
- How have the organisations that the participants work for supported CYP with long COVID?
  - Explore re both healthcare settings
  - Where participants haven’t had experience with Long Covid, explore views on how this would/could be managed.
  - What could be put in place to help organisations to support CYP in the future, e.g. resources, policies, training?
- How could CYP/ parents be supported to manage symptoms outside for healthcare or school settings?
- Do CYP (or adults) with LC tend to re-consult? How are patients supported long-term?
- What have the challenges been in supporting CYP with Long COVID?
  - What has helped?
  - What else might help?
- Have participants had access to information about Long COVID in CYP and appropriate training? What information or training would you find helpful?
